# Effects of Adalimumab, an Anti-tumour Necrosis Factor-Alpha (TNF-α) Antibody, on Obese Diabetic Rats

**DOI:** 10.21315/mjms2018.25.4.5

**Published:** 2018-08-30

**Authors:** Halima Ali Shuwa, Muhammad Kabiru Dallatu, Muhammed Haruna Yeldu, Hamidu Marafa Ahmed, Idris Abdullahi Nasir

**Affiliations:** 1 Department of Immunology, Faculty of Medical Laboratory Science, Usmanu Danfodiyo University, Sokoto, Nigeria; 2 Department of Chemical Pathology, Faculty of Medical Laboratory Science, Usmanu Danfodiyo University, Sokoto, Nigeria; 3 Department of Medical Laboratory Services, University of Abuja Teaching Hospital, Gwagwalada, FCT Abuja, Nigeria

**Keywords:** anti-inflammation, adalimumab, hyperglycemia, IL-6

## Abstract

**Background:**

Diabetes mellitus (DM) represents a major health problem worldwide. Recent studies have confirmed that obesity is a state of chronic inflammation that is characterised by increased concentrations of tumour necrosis factor-alpha (TNF-α), interleukin-6 (IL-6) and other inflammatory markers. It has been reported that increased TNF-α and IL-6 cause an immunological disturbance in DM. In the present study, the levels of fasting glucose, TNF-α and IL-6 were estimated in order to determine whether adalimumab can improve the glucose levels in obese diabetic rats.

**Materials and methods:**

Twenty-eight Wistar rats were divided into four groups: obese + diabetes + adalimumab (group 1), obese + diabetes (group 2), obese (group 3) and normal control (group 4), respectively (*n* = seven per group). Obesity was induced by feeding the rats in groups 1, 2 and 3 with a high-fat diet for four weeks. Some 30 mg/kg of streptozotocin (STZ) was administered to groups 1 and 2 so as to induce diabetes. Adalimumab was administered at a rate of 50 mg/kg to group 1 following the induction of diabetes. The fasting glucose, TNF-α and IL-6 concentrations were determined.

**Results:**

A significant decrease was observed in the glucose levels of the treated rats (6.91 [0.11] mmol/L) when compared to those of the untreated rats (15.43 [0.44] mmol/L) (*P* < 0.001). The TNF-α levels were lower in group 1 (20.71 [0.35] ng/L) than in groups 2 (37.90 [0.27] ng/L) and 3 (25.89 [0.12] ng/L) (*P* < 0.001), although they were higher when compared to the levels seen in group 4 (12.44 [0.38] ng/L) (*P* < 0.001). The IL-6 concentrations were found to be elevated in groups 1 (22.89 [0.45] ng/L), 2 (21.00 [0.40] ng/L) and 3 (31.80 [1.32] ng/L) when compared to the levels seen in group 4 (18.70 [0.37] ng/L) (*P* < 0.001), although they were lower in group 1 (22.89 [0.45] ng/L) than in group 3 (31.80 [1.32] ng/L) (*P* < 0.001).

**Conclusion:**

Adalimumab reduced the glucose and TNF-α levels of diabetic rats, which indicates that it has a therapeutic effect in terms of controlling the blood glucose.

## Introduction

Diabetes mellitus (DM), perhaps more commonly referred to as diabetes, is a group of metabolic diseases in which high blood sugar levels are observed over a prolonged period. The worldwide prevalence of DM was estimated to be 171 million people in 2010, and it is projected to increase to 366 million people by 2030 (1). In Nigeria, the prevalence of DM has been increasing steadily since 1971, although the reported prevalence values have not been uniform (i.e., they vary with different tribes, cultures and food values). However, in its current Diabetes Atlas, the International Diabetic Federation has recorded the prevalence of DM as 3.9% (5.5 million people), which positions Nigeria among those countries with a very high prevalence of diabetes (2).

DM is a serious and growing health concern worldwide, and it is associated with severe, acute and chronic complications that negatively influence both the quality of life and the survival of affected individuals. Recent studies have confirmed that obesity is a state of low-grade chronic inflammation that is characterised by increased concentrations of C-reactive protein (CRP), tumour necrosis factor-alpha (TNF-α), interleukin-6 (IL-6), and other inflammatory markers in the blood (3). The level of insulin resistance is known to be associated with the increased occurrence of myocardial infarction, stroke and peripheral vascular disease (4). Further, the levels of pro-inflammatory cytokines have been found to be increased in patients with type II diabetes mellitus (T2DM) (5).

TNF-α alters insulin sensitivity by means of altering different key steps involved in the insulin signaling pathway (6). The activation of pro-inflammatory pathways following exposure to TNF-α and IL-6 induces a state of insulin resistance in terms of the glucose uptake in the myocytes and adipocytes, which impairs insulin signaling at the level of the insulin receptor substrate proteins, subsequently resulting in hyperglycemia (7). Increased TNF-α and IL-6 production has been observed in adipose tissue taken from obese rodent or human subjects, and they have also been implicated as causative factors in obesity-associated insulin resistance and the pathogenesis of T2DM (8).

TNF-α is an adipocytokine involved in systemic inflammation that stimulates the acute phase reaction (9). TNF-α is primarily secreted by the macrophages, although it is also secreted by a wide variety of other cells, including the adipocytes (10). TNF-α inhibits insulin transduction, and it has an impact on glucose metabolism (11). Disturbances in the metabolism of TNF-α have been implicated in various metabolic disorders, such as obesity and insulin resistance (12), indicating that changes in the metabolism of TNF-α may affect the onset of T2DM, as well as the progression of the disease. IL-6 is an interleukin that acts as both a pro-inflammatory cytokine and an anti-inflammatory cytokine.

High levels of both IL-6 and TNF-α have been reported to be associated with insulin resistance in the adipocytes, hepatocytes and myocytes (13–15). Further, raised serum IL-6 and TNF-α levels in T2DM patients were found to be associated with an increased body mass index (BMI) (16). In a prospective study involving American females, the baseline CRP and IL-6 levels were found to be significantly higher in those who later developed T2DM (17).

Obesity has both genetic and environmental causes. It has a strong influence on the development of T2DM. Emerging evidence indicates that T2DM is a chronic inflammatory disease in which increased levels of cytokines are produced under various stimuli, such as overnutrition, increasing age and genetic or fetal metabolic preprogramming (18, 19). The associated chronic inflammation will result in glucose intolerance, diabetes and eventually, diabetic complications.

In addition to T2DM, obesity has also been reported to be associated with a systemic yet chronic inflammatory response characterised by altered cytokine production and the activation of inflammatory signaling pathways. Indeed, many reports have demonstrated the linkage between the increased production of inflammatory cytokines, such as TNF-α, IL-6 and certain adipokines, during the inflammatory process in obesity and the development of insulin resistance (20–22).

Anti-tumour necrosis factor (anti-TNF) drugs are a class of drugs used worldwide to treat inflammatory conditions. The currently available TNF-α inhibitors (etanercept, infliximab, adalimumab, certolizumab pegol and golimumab) aim to block the pro-inflammatory actions of this cytokine (23). Interestingly, the TNF-α inhibitors may also play a role in glycemic control, since the TNF-α molecule is known to affect glucose homeostasis. Hence, the outcomes affecting glycemic control may represent a side effect that is currently underpublicised in the literature. The evidence linking inflammation and DM dates back more than a century. Studies involving mice have shown positive correlation between the quantity of TNF-α and insulin resistance (24). Additionally, this finding has been confirmed in studies involving humans, including those with and those without T2DM (25).

Adalimumab is a fully humanised immunoglobulin G1 (IgG1) monoclonal antibody to TNF-α that has been approved for the treatment of rheumatoid arthritis psoriatic arthritis, and psoriasis (26). It binds to the soluble and transmembrane forms of TNF-α with high affinity, thereby preventing the TNF-α from binding to its receptors (27). Adalimumab (Humira; Abbott Laboratories) was originally genetically engineered using phage display technology, and it is structurally and functionally analogous to naturally occurring human IgG1 (28). The mechanism of action of adalimumab, as explained above, involves blocking the interaction of TNF-α with the p55 and p75 TNF-α cell surface receptors (28).

As recent studies have indicated that agents capable of improving insulin sensitivity may be of great value in the treatment of T2DM, it is highly important to investigate whether insulin resistance and its clinical correlates can be reversed using therapies aimed at the neutralisation of TNF-α.

The aim of the present study is therefore to assess the effect of adalimumab, an anti-TNF-α drug, on the glucose and TNF-α levels in streptozotocin (STZ)-induced diabetic rats.

## Materials and Methods

### Experimental Animals

Twenty-eight male Albino Wistar rats weighing 120–140 g and aged 8–12 weeks were purchased from the Animal House, Faculty of Pharmaceutical Sciences, Usmanu Danfodiyo University (UDUS), Sokoto, Nigeria. The animals were housed in standard cages during the months of July and August 2016, and they were acclimatised on normal diets for one week before they were fed with the experimental diets. All the animals were kept at a constant room temperature (25 °C–30 °C), in a 12/12 h light/dark cycle, with free access to food and water.

### Ethical Considerations

Ethical approval for the present study was obtained from the ethical review board of UDUS (UDUS/HREC/2016/NO.289).

### Study Design

This study is an experimental animal research study. Twenty-eight male Wistar rats were randomly assigned to one of four groups as follows:

Group 1: Obese, diabetic rats treated with 50 mg of adalimumab (*n* = 7).
Group 2: Obese, diabetic rats untreated (*n* = 7).
Group 3: Obese, non-diabetic rats serving as obese controls (*n* = 7).
Group 4: Non-obese, non-diabetic rats serving as controls (*n* = 7).

### Induction of Obesity

The experimental obesity condition was achieved by placing the rats in groups 1, 2 and 3 on a high-fat diet with a total kcal value of 40 kJ/kg (20% fat, 45% carbohydrate and 22% protein), which was prepared by mixing 40% of the normal diet with 60% of groundnut cake (29). The rats in the control group (group 4) were fed with a regular chow diet, which was sourced from Grand Cereals and Oil Mills Limited (Jos, Nigeria) throughout the study. The rats in all the groups were maintained on their diets for four weeks.

### Induction of Diabetes

T2DM was induced according to the method proposed by Zhang et al. (30). The rats in groups 1 and 2 were treated with a low dose (30 mg) of STZ (Sigma-Aldrich, St. Louis, MO). A single low dose of STZ (30 mg/kg, dissolved in normal saline) was intraperitoneally (IP) injected into each rat following 12 h of fasting. The control rats (groups 3 and 4) received a similar injection of just normal saline. Then, a 5% glucose solution was used as their drinking water for 24 h.

#### Inclusion and exclusion criteria

Some 72 h after the STZ injection, the rats were fasted overnight, and their fasting blood glucose levels were estimated using the Accu-Chek rapid test. Only those rats from groups 1 and 2 that exhibited a fasting blood glucose level of ≥ 7.1 mmol/L (≥ 126 mg/dl) were included in the study for further treatment.

### Treatment

The following treatment was administered following the induction of diabetes. Adalimumab (obtained from Humira; Abbott Laboratories, Abbott Park, IL; accession number: BTD00049) was simultaneously IP administered at a rate of 50 mg/kg to rats in group 1 on day 3 and day 10 (i.e., a week apart) following diabetes induction. This dosage was recommended by Mustafa et al*.* (31). The rats in groups 2, 3 and 4 received a similar injection of just normal saline. Samples were collected 48 h after the second adalimumab injection.

### Sample and Data Collection

On the last day of treatment, the rats were fasted overnight and then anaesthetised by dropping each rat into a transparent plastic jar saturated with chloroform vapour. Blood samples were obtained by means of cardiac puncture and then collected into fluoride oxalate and plain containers. The weights of the animals were measured using a weighing scale in grams (g), their lengths were measured with a measuring tape in centimeters (cm), while their BMIs (g/cm^2^) were calculated by dividing their weight (g) by the square of their length (cm^2^).

## Analytical Methods

### Estimation of Plasma Glucose

The plasma glucose levels of the rats were estimated using the oxidase-peroxidase method (32).

#### Principle of the test

Glucose oxidase (GOD) catalyses the oxidation of glucose in order to give hydrogen peroxide (H_2_O_2_) and gluconic acid. In the presence of the peroxidase (POD) enzyme, hydrogen peroxide is broken down, with the resultant oxygen reacting with both 4-aminophenazone (4-aminoantipyrine) and phenol to give a pink colour. The absorbance of the pink colour is directly proportional to the concentration of the glucose in the sample.

### Estimation of TNF-α

The serum TNF-α levels of the rats were estimated using the sandwich enzyme-linked immunosorbent assay (ELISA) method (supplied by WKEA Med Supplies Corp., Changchun, China).

#### Principle of the test

When using standard sandwich ELISA technology, in order to detect antigens, the wells of the microtiter plates are coated with a specific (capture) antibody, which is followed by incubation with the test solutions (serum) containing antigens. The unbound antigen is washed out and an antigen-specific antibody conjugated to the enzyme (i.e., developing reagent) is added, which is followed by another incubation. The unbound conjugate is washed out and a substrate is added. After another incubation, the degree of substrate hydrolysis is measured. The amount of the substrate that is hydrolysed is proportional to the amount of the antigen in the test solution. The generated calibration curve was used to extrapolate the concentration of the TNF-α in the specimen.

### Estimation of IL-6

The serum IL-6 levels of the rats were also quantified using the sandwich ELISA method (supplied by WKEA Med Supplies Corp., Changchun, China).

#### Principle of the test

When using standard sandwich ELISA technology, in order to detect antigens, the wells of the microtiter plates are coated with a specific (capture) antibody, which is followed by incubation with the test solutions (serum) containing antigens. The unbound antigen is washed out and an antigen-specific antibody conjugated to the enzyme (i.e., developing reagent) is added, which is followed by another incubation. The unbound conjugate is washed out and a substrate is added. After another incubation, the degree of substrate hydrolysis is measured. The amount of substrate that is hydrolysed is proportional to the amount of the antigen in the test solution. The generated calibration curve was used to extrapolate the concentration of the IL-6 in the specimen.

### Data Analysis

The data generated in this research study were analysed with the Statistical Package for the Social Sciences (IBM SPSS Statistics), version 24, using a one-way ANOVA and an ANCOVA. All data are presented as the mean (standard error of the mean [SEM]). When significant differences were observed, a post-hoc analysis was conducted in the form of a pair-wise comparison. A *P*-value of less than 0.05 (*P* < 0.05) was considered to be statistically significant.

## Results

The results obtained in this study are presented in Tables 1–4. The results depicted in Table 1 show a comparison of the means (SEM) of the anthropometric parameters of the obese + diabetes + adalimumab (group 1), obese + diabetes (group 2), obese (group 3) and normal control (group 4) rats. The mean (SEM) body weights (g) of the treated diabetic rats (306.86 [8.73] g), untreated diabetic rats (278.57 [4.29] g) and obese non-diabetic rats (307.29 [7.41] g) were significantly (*P* < 0.001) higher than the mean (SEM) body weight of the non-obese non-diabetic rats (193.71 [2.69] g). Similarly, a significant (*P* = 0.003) decrease in body weight was observed in the treated diabetic rats when compared to their untreated diabetic counterparts.

Moreover, the mean (SEM) BMIs (g/cm^2^) of the treated diabetic rats (0.563 [0.021] g/cm^2^), untreated diabetic rats (0.516 [0.015] g/cm^2^) and obese non-diabetic rats (0.554 [0.013] g/cm^2^) were significantly (*P* < 0.001) higher than the BMI of the non-obese non-diabetic rats (0.369 [0.005] g/cm^2^). Further, there was again a significant (*P* = 0.003) decrease in the body weights of the treated diabetic rats when compared to their untreated diabetic counterparts.

Table 2 demonstrates the diabetogenic effect of STZ in terms of inducing experimental DM in rats, as well as the effect of adalimumab on the plasma glucose levels of the treated, untreated, obese and non-obese rats. The results concerning the mean (SEM) fasting blood glucose concentrations (mmol/L) where significantly (*P* < 0.001) higher in the treated diabetic rats (6.91 [0.11] mmol/L), untreated diabetic rats (15.43 [0.44] mmol/L) and obese non-diabetic rats (8.07 [0.17] mmol/L) when compared to the concentration of the non-obese non-diabetic control rats (4.85 [0.35] mmol/L).

A statistically significant difference (*P* < 0.001) was found in terms of the fasting blood glucose level of the untreated diabetic rats when compared with the adalimumab-treated rats. Correspondingly, a significant difference was observed in the fasting blood glucose levels of the adalimumab-treaded and untreated diabetic rats when compared to the obese non-diabetic rats (*P* = 0.004 and *P* < 0.001, respectively).

A comparison of the mean (SEM) serum TNF-α concentrations (ng/L) of the groups is presented in Table 3. The mean (SEM) TNF-α concentrations of the adalimumab-treated diabetic rats (20.71 [0.35] ng/L), untreated diabetic rats (37.90 [0.27] ng/L) and obese non-diabetic rats (25.89 [0.12] ng/L) were significantly (*P* < 0.001 each) higher than the concentration of the non-obese non-diabetic control rats (12.44 [0.38] ng/L).

Similarly, the mean (SEM) of the TNF-α concentration was significantly lower (*P* < 0.001) in the adalimumab-treated rats than in the untreated diabetic rats and obese non-diabetic rats. Additionally, a significantly higher (*P* < 0.001) TNF-α concentration was observed in the untreated diabetic rats when compared to the obese non-diabetic rats.

Table 4 shows the mean (SEM) serum concentrations of IL-6 (ng/L) among the groups. The mean (SEM) IL-6 concentrations of the adalimumab-treated diabetic rats (22.89 [0.45] ng/L), untreated diabetic rats (21.00 [0.40] ng/L) and obese non-diabetic rats (31.80 [1.32] ng/L) were significantly higher (*P* < 0.001, *P* < 0.04 and *P* < 0.001, respectively) than the concentration of the non-obese non-diabetic control rats (18.70 [0.37] ng/L).

Similarly, the mean (SEM) of the IL-6 concentration was significantly lower (*P* < 0.001) in the adalimumab-treated rats than in the obese non-diabetic rats. Additionally, a significantly lower (*P* < 0.001) IL-6 concentration was observed in the untreated diabetic rats when compared to the obese non-diabetic rats. However, no statistically significant difference (*P* > 0.05) was observed in the IL-6 concentration of the adalimumab-treated rats when compared with the concentrations of their diabetic untreated counterparts.

## Discussion

In the present study, the effect of diabetes mellitus and the administration of adalimumab on the body weights of rats were determined. All the experimental rats used weighed between 120 g and 140 g at the commencement of the study. The mean body weight value of the non-obese, non-diabetic control rats was significantly lower than that of their diabetic counterparts at the end of the study. Similarly, the mean BMI of the non-obese, non-diabetic rats was significantly different from that of the diabetic rats.

Dallatu et al*.* (33) reported the effect of DM on the body weight of rats, and they noted that the diabetic unsupplemented rats exhibited a lower body weight than the non-diabetic rats. They explained that polyphagia and polyuria with glycosuria are both hallmarks of the diabetic state, with these conditions and others contributing to the wastage typically seen in diabetic subjects. The mobilisation of body fats and proteins as alternative sources of energy, in addition to nitrogenous wastage, in the diabetic state might also contribute to the body weight depreciation (28). Dallatu et al*.* (33) further reported the beneficial effect of antioxidants on body weight, especially in DM.

In the present study, it was observed that the administration of adalimumab causes a significant decrease in the fasting blood glucose levels of treated rats when compared with the levels seen in untreated rats. Correspondingly, this study showed a significant increase in the fasting blood glucose levels seen in the untreated obese, diabetic rats when compared to those seen in adalimumab-treated obese diabetic rats. Furthermore, a significant difference was found in the mean fasting glucose concentrations between the untreated obese diabetic rats and the obese non-diabetic rats. Additionally, the findings of this study showed that the mean TNF-α concentration in the non-obese non-diabetic rats was significantly lower than the concentrations seen in the treated obese diabetic rats and the untreated diabetic rats. The increase in the mean TNF-α value was more marked in the untreated diabetic rats than in the treated diabetic rats. Similarly, the mean TNF-α concentration was observed to be significantly higher in the obese rats than in the non-obese control rats.

Jadwiga et al. (34) reported a similar finding in a case series study wherein none of their patients had a personal history of either type I or type II DM. On a molecular level, the TNF-α molecule appears to inhibit the insulin-mediated uptake of glucose in adipose tissue via the mechanism of down-regulating the GLUT4 receptor. This could lead to a state of insulin resistance, with a subsequent increase in blood glucose levels (35). It is possible that, in the animals presented here, the action of the TNF-α inhibitors brought about increased insulin sensitivity due to the blockade of TNF-α in the adipose tissue (36). Hence, this could represent a potential mechanism for the development of the low blood glucose readings observed in the present study.

The evidence linking inflammation and DM dates back more than a century. For instance, one report showed that chronic treatment with adalimumab for either rheumatoid arthritis or psoriatic arthritis brought about a substantial improvement in insulin sensitivity (37). In the same study, one patient with T2DM actually reverted to a state in which glucose tolerance was impaired and insulin therapy ceased. Another prior study suggested that glycemic control could improve after 24 months of etanercept therapy (38). It stands to reason, therefore, that the TNF-α inhibitors used to treat rheumatoid arthritis could indeed induce a state of hypoglycemia following chronic use.

The first evidence that adipocytes obtained from obese animals express markedly increased amounts of TNF-α was presented by Hotamisligil et al. (39). Later data have shown that TNF-α is also expressed in human adipose tissue, and further, that its plasma concentration in obese subjects is decreased following weight loss (40). According to Zahran et al. (41), there is a significant positive correlation between TNF-α and BMI. Moreover, in-vitro studies of human cell lines have confirmed that, when exposed to TNF-α, adipocytes become insulin resistant (42). Mishima et al. (5) found that the level of TNF-α in obese T2DM patients depends on the degree of their insulin resistance, but not on their BMI. Finally, in addition to several indirect lines of evidence, the data derived from this study suggest that TNF-α plays a role in the pathophysiology of T2DM.

Additionally, the findings of this study demonstrated a relationship between the BMI and the TNF-α level in which there is a significant increase in the serum TNF-α concentration with an increase in the BMI, which is in agreement with the findings of the earlier studies by Gwozdziewiczova et al. (43) and Mishima et al. (5). Bertin et al*.* (44) detected correlation between the TNF-α level and the BMI with indices of intra-abdominal fat tissue, but not with glycaemia or the total amount of fatty mass in the body. Mishima et al. (5) found that the serum TNF-α concentration in obese individuals with T2DM depends on the degree of their insulin resistance, although it does not depend on their BMI.

The results of the present study demonstrated that the circulating TNF-α levels were significantly elevated in those with T2DM when compared with the normal healthy subjects, particularly in the case of obese subjects, and that it is also related to the BMI. Such data provide associative evidence supporting subclinical inflammation as a unifying factor that accelerates the progression of insulin resistance, as well as both the consequent glucose elevation and the development of T2DM. Data derived from this study hence suggest the possible role of TNF-α in the pathophysiology of T2DM, particularly in obese individuals.

The main finding of this study revealed significantly higher levels of IL-6 in the diabetic and obese groups than among the control subjects. Similar to previously reported studies (43, 45, 46), the present results showed an increased inflammatory status in type 2 diabetes. Previous studies have also shown that the circulating levels of IL-6 are high in T2DM patients (43, 45). Similar findings were reported in the present study. The in-vivo and in-vitro effects of IL-6 were studied by Bastard et al. (46) in relation to glucose uptake and insulin resistance. They concluded that IL-6 might be involved in the development of both increased blood glucose and insulin resistance (46). Rotter et al. (47) identified a similar relationship between IL-6 and insulin resistance, in which the IL-6 impairs the insulin signaling pathway in hepatocytes. According to another study, the time- and dose-dependent exposure of skeletal muscles to IL-6 may alter the insulin sensitivity of those skeletal muscles, which may eventually result in an elevation of the blood glucose (48).

The determination of the serum IL-6 level can be used to identify diabetic patients with a high risk of experiencing future cardiac events, since it stimulates the production of C-reactive protein (49). Ridker et al. (49) studied inflammatory signaling, and they suggested IL-6 as the marker of endothelial damage in elderly men. According to some research groups, IL-6 is simply an inflammatory marker that can determine the systemic inflammatory status. However, taking into account the pleiotropicity of this cytokine, as well as its wide array of actions on various cell types, the high inflammatory signaling mechanisms may precipitate the existing systemic and local inflammatory mechanism.

It has been suggested that T2DM is a disease of the innate immune system responsible for an ongoing cytokine-mediated acute phase response and low-grade chronic inflammation, which may be involved in both atherosclerosis and diabetes mellitus (51). Therefore, it seems important to determine whether the signs of an activated innate immune system are present prior to the onset of T2DM. Epidemiological evidence suggests that inflammatory markers, such as TNF-α and IL-6, predict the development of diabetes and glucose disorders (51). Indeed, TNF-α contributes to the pathogenesis of insulin resistance, T2DM, and abnormal adiposity or lipid disorders (9).

## Conclusion

Based on the findings of this study, the utilised anti-TNF-α agent (adalimumab) reduced the blood glucose levels in diabetic rats. This indicates that it has therapeutic potential in terms of controlling blood glucose and TNF-α. However, additional studies are required to substantiate this effect. An increase in the circulating TNF-α and IL-6 concentrations is associated with increased plasma glucose, body weight and BMI, which supports the idea that inflammatory cytokines play a role in the pathogenesis of diabetes and obesity. The results of this study showed higher concentrations of serum IL-6 in the diabetic group when compared with the controls. This suggests that IL-6, as an inflammatory mediator, is responsible for some underlying changes that contribute to the development of diabetes.

## Recommendations

Further investigations concerning the relationship between TNF-α, IL-6 and glucose equilibrium are warranted in human subjects.

## Figures and Tables

**Table 1:** Comparison of the mean (SEM) anthropometric parameters among the controls, treated and untreated rats (groups 1–4)

Groups	Weight (g)	Length (cm)	BMI (g/cm^2^)
Group 1 (*n* = 7)	306.86 (8.73)	23.34 (0.15)	0.563 (0.021)
Group 2 (*n* = 7)	278.57 (4.29)	23.27 (0.27)	0.516 (0.015)
Group 3 (*n* = 7)	307.29 (7.41)	23.60 (0.24)	0.554 (0.013)
Group 4 (*n* = 7)	193.71 (2.69)	22.29 (0.12)	0.369 (0.005)
F statistic	73.442	1.712	38.878
*P*-value	< 0.001	0.191	< 0.001
**Post-hoc using a pair-wise comparison**			
**Group comparison**	***P-*values**	***P-*values**	***P-*values**
Group 1 versus Group 2	0.003	0.910	0.023
Group 1 versus Group 3	0.921	0.284	0.602
Group 1 versus Group 4	< 0.001	0.199	< 0.001
Group 2 versus Group 3	0.004	0.292	0.061
Group 2 versus Group 4	< 0.001	0.225	< 0.001
Group 3 versus Group 4	< 0.001	0.146	< 0.001

Values are mean (SEM); *n* = number of subjects; group 1 = obese, diabetic, treated rats; group 2 = obese, diabetic, untreated rats; group 3 = obese, non-diabetic, control rats; group 4 = non-obese, non-diabetic, control rats

**Table 2:** Levels of fasting blood glucose (FBG) in the STZ-induced diabetic rats treated with adalimumab when compared with the controls

Groups	Fasting blood glucose (mmol/L) Mean (SEM)
Group 1 (*n* = 7)	6.91 (0.11)
Group2 (*n* = 7)	15.43 (0.44)
Group 3 (*n* = 7)	8.07 (0.17)
Group 4 (*n* = 7)	4.85 (0.35)
*F* statistic	330.567
*P*-value	< 0.001
**Post-hoc using a pair-wise comparison**	
**Group Comparison**	***P*-values**
Group 1 versus Group 2	< 0.001
Group 1 versus Group 3	0.004
Group 1 versus Group 4	< 0.001
Group 2 versus Group 3	< 0.001
Group 2 versus Group 4	< 0.001
Group 3 versus Group 4	< 0.001

Values are mean (SEM); *n* = number of subjects; group 1 = obese, diabetic, treated rats; group 2 = obese, diabetic, untreated rats; group 3 = obese, non-diabetic, control rats; group 4 = non-obese, non-diabetic, control rats

**Table 3:** Serum concentrations of TNF-α in the STZ-induced diabetic rats treated with adalimumab when compared with the controls

Groups	TNF-α (ng/L)
Group 1 (*n* = 7)	20.71 (0.35)
Group 2 (*n* = 7)	37.90 (0.27)
Group 3 (*n* = 7)	25.89 (0.12)
Group 4 (*n* = 7)	12.44 (0.38)
F statistic	1280.034
*P*-value	< 0.001
**Post-hoc using a pair-wise comparison**	
**Group comparison**	***P*-values**
Group 1 versus Group 2	< 0.001
Group 1 versus Group 3	< 0.001
Group 1 versus Group 4	< 0.001
Group 2 versus Group 3	< 0.001
Group 2 versus Group 4	< 0.001
Group 3 versus Group 4	< 0.001

Values are mean (SEM); *n* = number of subjects; group 1 = obese, diabetic, treated rats; group 2 = obese, diabetic, untreated rats; group 3 = obese, non-diabetic, control rats; group 4 = non-obese, non-diabetic, control rats

**Table 4:** Serum IL-6 concentrations of the STZ-induced diabetic rats treated with adalimumab when compared with the controls

Groups	IL-6 (ng/L)
Group 1 (*n* = 7)	22.89 (0.45)
Group 2 (*n* = 7)	21.00 (0.40)
Group 3 (*n* = 7	31.80 (1.32)
Group 4 (*n* = 7)	18.70 (0.37)
*F* statistic	58.561
*P*-value	< 0.001
**Post-hoc using a pair-wise comparison**	
**Group comparison**	***P*-values**
Group 1 versus Group 2	0.073
Group 1 versus Group 3	< 0.001
Group 1 versus Group 4	0.001
Group 2 versus Group 3	< 0.001
Group 2 versus Group 4	0.040
Group 3 versus Group 4	< 0.001

Values are mean (SEM); *n* = number of subjects; group 1 = obese, diabetic, treated rats; group 2 = obese, diabetic, untreated rats; group 3 = obese, non-diabetic, control rats; group 4 = non-obese, non-diabetic, control rats

## References

[1] Ginter E, Simko V (2010). Diabetes type 2 pandemic in 21st century. Bratisl Lek Listy.

[2] Ofoegbu E, Chinenye S (2013). National Clinical Practice Guidelines for Diabetes Management in Nigeria.

[3] Gimeno RE, Kalman LD (2005). Adipose tissue as an active endocrine organ: recent advances. Current Opinion on Pharmacology.

[4] Misra A, Vikram NK (2002). Insulin resistance syndrome (metabolic syndrome) and Asian Indians. Curr Sci.

[5] Mishima Y, Kuyamna A, Tada A, Takahashi K, Ishioka T, Kibata M (2001). Relationship between TNF-alpha and insulin resistance ion obese men with type 2 diabetes mellitus. Diabetes Res Clin Pract.

[6] Bastard JP, Maachi M, Lagethu C, Kim MJ, Caron M, Vidal H (2006). Recent advances in the relationship between obesity, inflammation and insulin resistance. European Cytology Network.

[7] Iria NV, Sonia F, David K, Rocio VB, Lucia GG, Margareta L (2008). Insulin resistance associated to obesity: the link TNF-alpha. Arch Physiol Biochem.

[8] Aguirre VU, Chida TY, Enush L, Davis R, White MF (2000). The c-Jun NH2-terminal kinase promotes insulin resistance during association with insulin receptor substrate-1 and phsosphorylation of Ser^307^. J Biol Chem.

[9] Moller DE (2000). Potential role TNF-a in the pathogengsis of insulin resistance and type 2 diabetes trends. Endocrinal Metabolism.

[10] Giemeno RE, Klaman LD (2005). Adipose tissue as an actibe endocrine organ; recent advances. Current Opinion on Pharmacology.

[11] Zou C, Shao J (2008). Role of adipocytokines in obesity-associated insulin resistance. J Nutr Biochem.

[12] Groop LC, Saloranta C, Shank M, Bonadonna RC, Ferrannini E, DeFronzo RA (1991). The role of free fatty acid metabolism in the pathogenesis of insulin resistance in obesity and non-insulin dependent diabetes mellitus. J Clin Endocrinol Metab.

[13] Hoene M, Weigert C (2008). Role of interleukin-6 in insulin resistance, body fat distribution and energy balance. Obesity Review.

[14] Glunds S, Krook A (2008). Role of interleukin-6 signaling in glucose and lipid metabolism. Acta Physiologica.

[15] Kristiansen OP, Mandrup-Poulsen T (2005). Interleukin 6 and diabetes: the good, the bad or the indifferent?. Diabetes.

[16] Testa R, Olivieri F, Bonfigli AR, Sirolla C, Boemi M, Marchegiani F (2006). Interleukin-6-174G > C polymorphism affects the association between IL-6 plasma levels and insulin resistance in type 2 diabetic patients. Diabetes Res Clin Pract.

[17] Hu FB, Meigs JB, Li TY, Rifai N, Manson JE (2004). Inflammatory markers and risk of developing type 2 diabetes in women. Diabetes.

[18] Dandona P, Aljada A, Bandyopadhyay A (2004). Inflammation: the link between insulin resistance, obesity and diabetes. Trends Immunol.

[19] Lumeng C, Maillard I, Saltiel A (2009). T-ing up inflammation in fat. Nat Med.

[20] Andersson CX, Gustafson B, Hammarstedt A (2009). Inflamed adipose tissue, insulin resistance and vascular injury. Diabetes Metab Research Review.

[21] Gustafson B, Hammarstedt A, Andersson CX (2007). Inflamed adipose tissue: a culprit underlying the metabolicsyndrome and atherosclerosis. Arterioscler Thromb Vasc Biol.

[22] Maury E, Brichard SM (2010). Adipokine dysregulation, adipose tissue inflammation and metabolic syndrome. Mol Cell Endocrinol.

[23] Muturu O, Laakso M, Isomaki H, Koota K (1989). Cardiovascular mortality in patients with arthritis. Cardiology.

[24] Kumeda Y, Inaba M, Goto H (2002). Increased thickness of the arterial intima media detected by ultrasonography in patients with rheumatoid arthritis. Arthritis Rheumatol.

[25] Park YB, AHNCW, Choi HK (2002). Atherosclerosis in rheumatoid arthritis. Morphologic evidence obtained by carotid ultrasound. Arthritis Rheumatol.

[26] Wong M, Ziring D, Korin Y (2008). TNF alpha blockade in human diseases: mechanisms and future directions. Clinical Immunology.

[27] Shen C, Assche G, Colpaert S (2005). Adalimumab induces apoptosis of human monocytes: a comparative study with infliximab and etanercept. Aliment Pharmacol Ther.

[28] Salfeld J, Kaymakcalan Z, Tracey D, Roberts A, Kamen R (1998). Generation of fully human anti-TNF antibody D2E7. Arthritis Rheumatol.

[29] Michael M, Chukwudike A, Phillips O (2015). Effects of high dietary fat intake on biochemical variables and pancreas histoarchitecture in diabetic rats. Journal Human Nutrition and Food Science.

[30] Zhang M, Lv XY, Li J, Xu ZG, Chen L (2008). The characterization of high-fat diet and multiple low-dose Streptozotocin induced type 2 diabetes rat model. Exp Diabetes Res.

[31] Mustafa Y, Selma T, Ozkan H, Ozlem O, Sima S, Tukay B (2010). Ameliorative effect of adalimumab on experimentally induced acute pancreatitis in rats. Pancreas.

[32] Trinder P (1969). Determination of glucose in blood using glucose oxidase with on alternative oxygen receptor. Ann Clin Biochem.

[33] Dallatu MK, Anaja PO, Bilbis LS, Mojiminiyi FB (2009). Antioxidant micronutrient potentials in strengthening the antioxidant defence in alloxan-induced diabetic rats. Niger J Pharm Sci.

[34] Jadwiga B, Czajkowska BS, Susan Z (2012). Development of low blood glucose readings in nine non-diabetic patients treated with tumor necrosis factor-alpha inhibitors. J Med Case Rep.

[35] Bonilla E, Lee YY, Phillips PE, Perl A (2001). Hypoglycemia after initiation of treatment with etanercept in a patient with type 2 diabetes mellitus. Ann Rheum Dis.

[36] Alberti KG, Zimmet PZ (2003). Definition, diagnosis and classification of diabetes mellitus and its complications. Part 1: diagnosis and classification of diabetes mellitus provisional report of a WHO consultation. Diabetes Medicine.

[37] Yazdani BB, Stelzl H, Brezinschek HP, Hermann J, Mueller T, Krippl P (2004). Improvement of insulin sensitivity in insulin resistant subjects during prolonged treatment with the anti-TNF-alpha antibody infliximab. Eur J Clin Invest.

[38] Mastrandrea L, Yu J, Behrens T, Buchlis J, Albini C, Fourtner S (2009). Etanercept treatment in children with new-onset type 1 diabetes: pilot randomized, placebocontrolled, doubleblind study. Diabetes Care.

[39] Hotamisligil GS, Shargill NS, Spiegelman BM (1993). Adipose expression of tumor necrosis factor-alpha: direct role in obesity-linked insulin resistance. Science.

[40] Hotamisligil GS, Arner P, Caro JF, Atkinson RL, Spiegelman BM (1995). Increasd adipose tissue expression of tumor necrosis factor-alpha in human obesity and insulin resistance. J Clin Invest.

[41] Zahran A, Enas S, Essa W, Waleed F, Abdul E (2012). Study of serum tumor necrosis factor alpha and interleukin 6 in type 2 diabetic patients with albuminuria. Life Sci J.

[42] Saghizadeh M, Ong JM, Garvey WT, Henry RR, Kern PA (1996). The expression of TNF-alpha by human muscle. Relationship to insulin resistance. J Clin Invest.

[43] Gwozdziewiczova S, Lichnovska R, Ben Y, Ahia R, Chlup R, Hrebicek J (2005). TNFα in the development of insulin resistance and other disorders in metabolic syndrome. Biomedical Papers of the Medical Faculty of the University of Palacky, Olmouc Czech Repub.

[44] Bertin E, Nguyen P, Guenounocu M, Durlach V, Potron G, Leutenegger M (2000). Plasma levels of tumor necrosis factor-alpha (TNF-alpha) are essentially dependent on visceral fat amount in type 2 diabetic patients. Diabetes Metab.

[45] Hotamisligil GS, Budavari A, Murray DL, Spiegelman BM (1994). Reduced tyrosine kinase activity of the insulin receptor in obesity-diabetes: central role of tumor necrosis factor-alpha. J Clin Invest.

[46] Bastard JP, Macchi M, Van N (2002). Adipose tissue IL-6 content correlates with resistance to insulin activation of glucose uptake both in vivo and in vitro. J Clin Endocrinol Metab.

[47] Rotter V, Nagaev I, Smith U (2003). Interleukin-6 (IL-6) induces insulin resistance in 3T3-L1 adipocytes and is, like IL-8 and tumor necrosis factor-alpha, over expressed in human fat cells from insulin-resistant subjects. J Biol Chem.

[48] Mooney R (2007). Counterpoint: interleukin-6 does not have a beneficial role in insulin sensitivity and glucose homeostasis. J App Physiol.

[49] Ridker PM, Wilson PW, Grundy SM (2004). Should C–reactive protein be added to metabolic syndrome and to assessment of global cardiovascular risk?. Circulation.

[50] Fernandez-Real JM, Ricart W (2002). Insulin resistance and chronic cardiovascular inflammatory syndrome. Endrocrine Review.

[51] Schmidt MI, Duncan BB, Sharrett AR, Lindberg G, Savage PJ, Offenbacher S (1999). Markers of inflammation and prediction of diabetes mellitus in adults (atherosclerosis risk in communities study): a cohort study. Lancet.

